# Ayurvedic Herbal Medicines: A Literature Review of Their Applications in Female Reproductive Health

**DOI:** 10.7759/cureus.55240

**Published:** 2024-02-29

**Authors:** Srihita Patibandla, Joshua J Gallagher, Laasya Patibandla, Ali Z Ansari, Shayaan Qazi, Samuel F Brown

**Affiliations:** 1 Obstetrics and Gynecology, William Carey University College of Osteopathic Medicine, Hattiesburg, USA; 2 Obstetrics and Gynecology, Western University, London, CAN; 3 Obstetrics and Gynecology, University of South Florida, Tampa, USA; 4 Obstetrics and Gynecology, South Central Regional Medical Center, Laurel, USA

**Keywords:** fertility inducing drugs, lactation, polycystic ovary syndrome (pcos), primary dysmenorrhea, laparoscopic surgery for endometriosis, nausea and vomiting in pregnancy, herbal medicines, female reproductive health, ayurveda, obstetrics and gynecology

## Abstract

Ayurveda, an ancient holistic and personalized healing system originating from the Indian subcontinent, has been gaining increasing attention as a complementary and alternative medical practice for treating various health conditions, including those related to women’s reproductive well-being. This comprehensive literature review examines a wide array of experimental and clinical studies exploring the diverse facets of Ayurvedic interventions in addressing issues such as menstrual irregularities, polycystic ovary syndrome (PCOS), infertility, and menopausal symptoms. The paper specifically focuses on discussing the available data regarding the efficacy of Tulsi (*Ocimum tenuiflorum*), ashwagandha (*Withania somnifera*), ginger (*Zingiber officinale*), cardamom (*Elettaria cardamomum*), turmeric (*Curcuma longa*), and Shatavari (*Asparagus racemosus*)*, *which have traditionally been used in Ayurvedic medicine for centuries. The synthesis of literature not only highlights the potential benefits of these Ayurvedic interventions, but also critically assesses the methodological rigor of existing studies, identifying research gaps, and proposing directions for future investigations. While acknowledging the need for further rigorous research and clinical trials, the review emphasizes the benefits of collaborative and integrative healthcare. This review aims to serve as a valuable resource for healthcare practitioners, researchers, and individuals seeking holistic and natural alternatives for female reproductive health management.

## Introduction and background

In its pursuit of comprehensive well-being and longevity, humanity has always looked towards ancient wisdom, and at the forefront of this timeless knowledge stands Ayurveda. The word Ayurveda, derived from the Sanskrit words 'Ayus' (meaning all aspects of life from birth to death) and 'Veda' (meaning knowledge), translates to "the science of life" [1]. Emphasizing a holistic approach to life, health, and disease management through the use of natural remedies, diet, lifestyle changes, and spirituality, Ayurveda aims to not only cure illness but also prevent diseases by maintaining health and promoting longevity [1]. Its foundational knowledge, found in texts such as the Charaka Samhita, Sushruta Samhita, and Ashtanga Hridaya, encompasses intricate details of over 700 herbs and 6,000 formulations, providing a comprehensive understanding of various diseases, diagnostic methods, and practical dietary and lifestyle recommendations [2]. These ancient manuscripts serve as a rich source of wisdom for holistic health and well-being. One of the cornerstones of Ayurveda is its profound emphasis on the therapeutic potential of herbal medicine [3]. In India, around 20,000 medicinal plants have been recorded, with practitioners of traditional medicine using around 7,000-7,500 of these plants for the treatment of various medical conditions [3].

In the domain of female reproductive health, the branches of Ayurveda known as “Prasuti Tantra” (obstetrics) and “Stri Roga” (gynecology) focus on promoting women’s well-being through proper nutrition, prevention of disease, and specialized treatments for various medical illnesses [4]. In the Ayurvedic philosophy, Dosha (defects), Dhatu (elements or tissues of the body), and Mala (waste products) are said to be the primary components of the human body that can be influenced through herbal remedies and lifestyle modifications [4]. Vata Dosha ("Vata" translates to "space and air") is associated with movement, and underlying processes such as breathing, circulation, and elimination of wastes. In female reproductive health, Vata Dosha would thus influence physiological and pathologic processes such as menstrual abnormalities, dysmenorrhea, chronic pelvic pain, infertility, post-menopausal symptoms, vaginal dryness, dyspareunia, and developmental anomalies of the genital tract. Alternatively, Pitta Dosha ("Pitta" translates to "fire and water") is associated with processes such as heat, metabolism, and transformation, manifesting itself in women’s reproductive processes such as vaginal bleeding, premenstrual syndrome, infertility, abortion, hormonal imbalance, inflammatory conditions, pelvic inflammatory disease, urinary tract infections, and abnormal uterine bleeding. Finally, the third component of Dosha known as Kapha Dosha ("Kapha" translates to "earth and water") is reported to influence the time from childhood up until puberty and is associated with characteristics such as softness and immaturity. In women’s reproductive health, Kapha Dosha underlies pathologies such as vulvovaginitis, obesity, delayed puberty, amenorrhea, yeast infections, benign tumors, fibrocystic breast and ovaries, infertility, menstrual irregularities, and polycystic ovary syndrome (PCOS). Treating imbalances in these three Doshas has been associated with positive changes in treating disorders related to women’s health [4].

In the field of obstetrics, the incorporation of herbal interventions has become an intriguing pathway, presenting nuanced solutions that specifically target common aspects. These encompass menstrual health, fertility support, general well-being, conception, pregnancy, and childbirth [1]. The integration of these herbal interventions offers a comprehensive approach to women's reproductive health, acknowledging the multifaceted nature of these interconnected elements. The rich tradition of Ayurveda encompasses a diverse array of herbs, each chosen for its unique properties to support overall well-being. This literature review aims to unravel the therapeutic potential of various herbs, spotlighting the roles of Tulsi (*Ocimum tenuiflorum*), ashwagandha (*Withania somnifera*), ginger (*Zingiber officinale*), cardamom (*Elettaria cardamomum*), turmeric (*Curcuma longa*), and Shatavari (*Asparagus racemosus*) in promoting female reproductive wellness. These botanical agents have been traditionally employed to address an array of reproductive health concerns, ranging from dysmenorrhea, PCOS, and infertility to the management of symptoms associated with pregnancy, such as nausea and vomiting [4,5]. The holistic approach embedded in Ayurveda, coupled with scientific investigations into the bioactive compounds within these herbs, warrants a nuanced understanding of their applications in diverse reproductive health contexts.

The integration of Ayurvedic practices into modern medical systems presents an interesting intersection of traditional and modern medicine. Ayurveda's holistic approach to well-being, its individualized approach, and natural remedies align with the growing interest in personalization and patient-centered care. However, Ayurveda's rich tradition and diverse treatments may not always align with the rigorous standards of evidence-based modern medicine. The lack of well-designed, large-scale clinical trials makes it challenging to establish the safety, efficacy, and specific mechanisms of these interventions for various health conditions. This scarcity of comprehensive research poses a challenge to Ayurveda gaining wider acceptance and integration into mainstream healthcare. Additionally, variations in formulations, treatment modalities, and practitioner approaches within Ayurveda make it difficult to standardize and incorporate into mainstream healthcare. On the positive side, Ayurveda's emphasis on preventative healthcare complements modern medicine, contributing to a more comprehensive healthcare model where both healthcare systems can be used in conjunction. The potential synergistic effects between the two fields enable enhanced treatment outcomes, especially in the management of chronic conditions such as PCOS and endometriosis. This article aims to summarize the latest information and research related to Ayurvedic herbs, offering a comprehensive overview of their contributions to female reproductive health. While recognizing the value of Ayurveda as a potential alternative approach to healthcare, this article highlights the importance of collaboration between traditional therapies and modern medical practices.

## Review

Methodology 

We searched the databases PubMed and Google Scholar by using the following keywords in different combinations: “Tulsi", "Ashwagandha", "Shatavari", "*Asparagus racemosus*", "Cardamom", "Ginger", "Turmeric", "Curcumin", "*Ocimum tenuiflorum*", "*Withania somnifera*", "*Elettaria cardamomum*", "*Zingiber officinale*", "*Curcuma longa*", "PCOS", "female reproductive health", "pregnancy", "dysmenorrhea", "infertility", "contraception", "endometriosis", "menstruation", "women's health", and "Ayurveda" (last search conducted in January 2024). In the present literature review, we focused our attention on herbs that are frequently used for the most common conditions affecting women's health including (1) herbs used to treat PCOS, (2) herbs used to counteract pregnancy-related nausea and vomiting, (3) herbs used to treat dysmenorrhea, (4) herbs used to treat infertility, (5) herbs used for contraception, and (6) herbs used to treat endometriosis. We included experimental data on animals, as well as clinical trials and surveys on women with these reproductive conditions. Finally, the inclusion criteria included observational and experimental studies that appeared to be the most interesting or relevant to the topic of our paper. Studies that were published in languages other than English were excluded.

Shatavari (*Asparagus racemosus*)

*Asparagus racemosus*, traditionally known as Shatavari, is a versatile herb widely used in traditional medicine like Ayurveda, Unani, and Siddha, for its potential benefits for women’s health [6]. Native to India and the Himalayas, *Asparagus racemosus* is renowned for its delicate, climbing vines and needle-like leaves [7]. The herb is characterized by tuberous roots that are harvested and dried for their medicinal properties [6,8]. It is known to contain bioactive compounds such as saponins, asparagamine, racemosol, polysaccharides, mucilage, folic acid, sarsasapogenin, flavonoids, and polyphenols [7,8]. These components are believed to impact hormonal balance, and have adaptogenic properties, helping the body adapt to stress and potentially alleviating symptoms associated with hormonal fluctuations. Consequently, *Asparagus racemosus* is used for immunity, preventing aging, cognitive functioning, neurologic disorders, dyspepsia, tumors, inflammation, neuropathy, and liver disease [8]. In female reproductive health, *Asparagus racemosus* is used for its phytoestrogenic properties in treating menopausal symptoms and enhancing lactation [8]. 

The rising interest in managing dysmenorrhea has fueled the exploration of natural remedies, driven by their potential efficacy and minimal side effects compared to conventional treatments. In an animal study by Kaaria et al. [9], the effect of *Asparagus racemosus* and ibuprofen on isolated uterine strips was evaluated using six nulliparous, non-pregnant female albino Wistar rats. Different concentrations of plant extract (20, 40, 80, and 160 mg/mL) were exposed to the uterine strips, with 20 mg/mL ibuprofen as a positive control. The authors observed a significant increase in the proestrus phase of the estrous cycle and a consequent decrease in the metestrus and diestrus phases with exposure to the plant extract. *Asparagus racemosus* was also shown to cause a dose-dependent reduction in uterine contraction force and frequency of contractions [9]. While these characteristics underscore its use in the management of dysmenorrhea, the very small sample size used renders the reproducibility of these findings problematic. 

Exploring the diverse application of *Asparagus racemosus*, in a study done by Kumar et al. [10] on Karan Fries crossbred cows, the effects of prepartum* Asparagus racemosus* root powder supplementation on milk production and reproductive performance, amongst other factors, was studied. Experimental group cows were supplemented with *Asparagus racemosus* powder at 100 mg/kg of their body weight every morning from 60 days until delivery, with non-supplemented cows as the control group. *Asparagus racemosus-*supplemented cows had faster delivery of placental membranes and greater milk yield in the post-partum period than controls [10]. The study, while suggesting efficacy in the use of the plant extract for boosting lactation in mothers, warrants further research involving greater sample sizes. However, in human studies, Gupta and Shaw [11] evaluated the galactogogue effects of *Asparagus racemosus *on lactating mothers by measuring the levels of their prolactin hormone. Secondary parameters such as mother and baby’s weights, and mother’s subjective reports of their satisfaction and their baby’s happiness were explored. *Asparagus racemosus* was found to significantly increase prolactin hormone levels by more than three-fold, with a positive association found with the secondary outcome measures [11]. The study reinforces the galactogogue effects of *Asparagus racemosus*, warranting its use in human populations.

Fertility-inducing medicines are gaining popularity in the modern era, with clomiphene citrate being used as the first-line treatment. In another randomized controlled trial by Majeedi et al. [12], 40 women were randomly divided into a test group where 6 grams of* Asparagus racemosus* powder was administered orally twice a day on days 1-14 of their menstrual cycle and a control group where 50 grams of clomiphene citrate was given orally once a day on days two to six of their cycle, both for two consecutive cycles. The effects of *Asparagus racemosus *were directly comparable to the gold standard, clomiphene citrate, in stimulating follicular growth (30% and 40% in the test group, 60% and 50% in the control group for the first and second cycles, respectively) and ovulation (25% and 30% in the test group, 40% and 25% in the control group for the first and second cycles, respectively) [12]. The herb holds promise as an alternative fertility treatment for women experiencing significant side effects with pharmaceutical therapies. 

Menopausal syndrome, another concern faced by aging women, is characterized by the cessation of menses in association with stress, anxiety, depression, hot flashes, headaches, and insomnia [13]. In 2015 a placebo-controlled randomized single-blind study by Farzan and Sultana [13], the efficacy of *Asparagus racemosus* for menopausal syndrome was studied in women aged 40 to 60 years. Test group patients were orally administered 3 grams of powder containing Glycyrrhiza glabra (licorice) and *Asparagus racemosus*, and control patients were administered 3 grams of roasted wheat flour twice daily for eight weeks. Patients treated with the herbs reported fewer hot flashes and night sweats over 24 hours, lower anxiety by the Hamilton anxiety scale, and lower insomnia by the Pittsburgh Sleep Quality Index Duration (PSQIDURAT) scale by the end of the trial [13]. Given that standard hormone replacement therapies (HRT) have a host of side effects, herbs like *Asparagus racemosus* prove to be noteworthy substitutes with their reported lack of toxicity and mortality as seen in a study by Bhandary et al. [14]. Table 1 presents the characteristics of the selected studies evaluating the use of *Asparagus* *racemosus *in female reproductive health.

**Table 1:** Characteristics of the research papers on the applications of Asparagus racemosus in female reproductive health

Research studies						
Reference	Subjects	Treatment	Study design	Sample size (n)	Adverse effects	Main results
Kaaria et al., 2019 [9]	Uterine strips from nulliparous nonpregnant female albino Wistar rats 4 months old	20, 40, 80, and 160 mg/mL of *Asparagus racemosus* extract for 10 minutes each	Experimental Study	6	None	Significant increase of proestrus phase (p<0.001), a significant reduction in the metestrus (p<0.01) and diestrus (p<0.05) with plant extract exposure than control. The plant extract caused a dose-dependent significant reduction in uterine force of contraction by (-0.15%, -5.13%, -7.97%, and -19.55%) at 20, 40, 80, and 160 mg/ml respectively. The plant extract also caused a significant decline in the frequency of uterine contraction (-5.99%; -9.61%; -16.76% and -25.21%). The extract caused no mortality even at the limited dose of 5000 mg/kg
Kumar et al., 2014 [10]	Healthy advanced pregnant crossbred Karan Fries cows	*Asparagus racemosus* root powder fed at 100 mg/kg of live body weight once in the morning from ~60 days until parturition	Experimental study	10	None	Milk yield was significantly higher (p<0.01) in the test group than in the control group. Colostrum protein, total solids, solids not fat (SNF) (p<0.05), and total immunoglobulin levels were higher (p<0.01) in the test group than in the control group. Cows of the test group took less time to expel placental membranes (p<0.05) and had less service period and service/conception (P<0.05) than the control group
Gupta and Shaw, 2011 [11]	Lactating mothers between age 20-40 with infant age up to 6 months	The test group received *A. racemosus* root powder (60 mg/kg of body weight daily), the control group received rice powder (60 mg/kg of body weight daily)	Double-blind randomized clinical trial	60	None	Over three-fold prolactin hormone elevation in the test group compared to the control group (p<0.05). A statistically significant positive difference between test and control groups in secondary outcomes of subjective mothers’ satisfaction and baby’s happiness (p<0.05)
Majeed et al., 2016 [12]	Married women between the ages of 18-40 years	6 g of *Asparagus racemosus* powder twice daily from day 1-14 of the cycle in the test group, clomiphene citrate 50 mg once daily from day 2-6 of the cycle was administered orally for 2 consecutive cycles	Single-blind randomized controlled trials	40	None	After the 1st and 2nd cycles, a strongly significant difference in follicle growth (>10 mm) was observed in the control group (p=0.007 and 0.006); the difference in follicle growth (>14 mm) was suggestively significant (p=0.093 and 0.052) in the control group and the difference in follicle growth (>17mm) was moderately significant (p=0.046) in the control group after 1st cycle of treatment
Farzana and Sultana, 2015 [13]	Women between the ages of 40 and 60 years of age with amenorrhea of more than one year duration	The test group was treated with 3 g of powder of Glycyrrhiza glabra and *Asparagus racemosus*, control group was treated with 3 g of roasted wheat flour twice a day for 8 weeks	Double-blind randomized controlled trial	60	None	The frequency of hot flashes and night sweats in 24 hours, anxiety by the Hamilton anxiety scale, and insomnia by the PSQIDURAT scale were significantly lower in the test group compared to the control group (p<0.001)

Cardamom (*Elettaria cardamomum*)

Cardamom, a flavorful spice derived from the seeds of plants in the Zingiberaceae family, has been a staple in culinary traditions for centuries [15]. Scientifically known as *Elettaria cardamomum*, the herb is native to the Indian subcontinent [15,16]. Rich in essential oils, cardamom contains compounds like terpenoids and flavonoids, contributing to its aromatic properties [16]. Additionally, cardamom also contains nutrients like potassium, calcium, and magnesium. The bioactive compounds in cardamom such as cineole, terpineol, terpene, and volatile oil exhibit antioxidant and anti-inflammatory properties, leading to the exploration of its impact on reproductive health [15,17]. 

One of the most common applications for cardamom in the realm of female reproductive health is in pregnancy. In a recent study, Sari et al. [18] explored cardamom ginger pudding’s efficacy in reducing the frequency of nausea and vomiting in 16 pregnant women in their first trimester. The majority of respondents experienced medium-level nausea and vomiting before receiving cardamom ginger pudding. After consumption, the majority reported mild symptoms and there was a statistically significant reduction in nausea and vomiting frequency [18]. However, with ginger’s well-established antiemetic properties, it serves as a confounding variable in this experiment and consequently, the study cannot speak on the effects of cardamom alone on nausea and vomiting [19]. The study also consisted of a small sample size, and its generalizability must be further explored. Another study looked into the effect of cardamom inhalation therapy on intra- and postoperative nausea and vomiting of mothers undergoing spinal anesthesia for elective cesarean section. This study by Khatiban et al. [20] had participants inhale, upon the first episode of nausea, through a plastic bag containing distilled gauze pads soaked in normal saline with or without cardamom essential oil. After treatment, both the placebo and cardamom groups experienced a reduction in nausea severity, with a more pronounced decrease observed in the cardamom group after adjusting for initial nausea severity. The cardamom group exhibited significantly lower rates of nausea episodes and retching compared to the placebo group. These findings highlight the potential effectiveness of cardamom in reducing nausea severity and associated symptoms, and incidentally also highlight the decreased need for antiemetic medications in comparison to the placebo [20]. More studies are needed to determine if inhalation therapy may be more effective in controlling emetic episodes as compared to ingested cardamon.

Several other studies have investigated the use of cardamom in women with polycystic ovary syndrome (PCOS). Cheshmeh et al. [21] studied inflammatory genes in 194 obese women with PCOS on a low-calorie diet. The test participants were given 3 g of green cardamom daily, compared to a placebo group that received 3 g of starch powder. Anthropometric indices such as weight, body fat mass, and waist circumference were significantly lower in both groups due to the low-calorie diet. Luteinizing hormone (LH), androstenedione, and dehydroepiandrosterone (DHEA) were significantly lower in the green cardamom group, and follicle-stimulating hormone (FSH) was higher. Inflammatory markers seen in polycystic ovary diseases such as TNF-α, IL-6, and C-reactive protein (CRP) were decreased in the serum in the green cardamom group compared to the placebo. The expression of TNF-α and c-reactive protein genes was also significantly decreased [21]. In another study by Cheshmet et al. [22] with similar setup parameters, the group looked further into the effects of cardamom on obesity and diabetes gene expression in obese women with PCOS. Here they discovered improved glycemic indices and lower levels of androgen hormones in the cardamom intervention group compared to the starch powder placebo. The obesity and diabetes genes fat mass and obesity-associated (FTO), carnitine palmitoyl transferase 1A (CPT1A), leptin receptor (LEPR), and lamin A/C (LAMIN) genes were significantly down-regulated in the cardamom group. The peroxisome proliferative activating receptor-γ (PPAR-γ) gene was up-regulated [22]. These studies demonstrate the potential benefits of cardamom in improving gene expression, hormonal levels, and inflammatory markers. The positive effects of gene expression related to obesity and diabetes further support the promising role of cardamom in managing PCOS-related complications [21,22].

While these studies show promise for the therapeutic roles of cardamom in women's health, the toxicity profile of cardamom is noteworthy. In a study by Masoumi-Ardakani et al. [23], cardamom extract and essential oil showed significant movement toxicity and neurotoxicity at doses of 1.5 g/kg and 0.75 g/kg in mice. However, no significant lethality was noted [23]. No adverse effects were reported by the participants in any of the other studies discussed in this section. Keeping this in mind, further research on the toxicity profile of long-term cardamom use is needed before the herb is considered for use in clinical practice. 

The findings of the selected studies on the medicinal use of cardamom are presented in Table 2.

**Table 2:** Characteristics of the research papers on the applications of cardamom in female reproductive health

Research studies						
Reference	Subjects	Treatment	Study design	Sample size (n)	Adverse effects	Main results
Sari et al., 2023 [18]	Pregnant women in their 1^st^ trimester	Cardamom ginger pudding was made with 5 cardamom pods, 250 mg ginger, 500 mL water, and 50 g sugar. Administered 2 times daily, 250 mL/1 glass once in the morning and once in the afternoon for 7 consecutive days	Quantitative study using the pre-experiment method research design with a pre-test and post-test	16	None	Before consuming the Cardamom ginger pudding, the frequency of nausea and vomiting was rated to be in the medium category by 62.6% of the women. After consuming the pudding, the frequency of nausea and vomiting was mostly in the mild category as rated by 68.7% of the women (p<0.05)
Khatiban et al., 2022 [20]	Mothers undergoing spinal anesthesia for elective cesarean section	Upon the first episode of nausea, participants inhaled through a plastic bag containing distilled gauze pads in normal saline with or without Cardamom essential oil	Single-blind randomized controlled trial	70	None	Following the intervention, nausea severity in placebo (25.28 ± 32.38) and cardamom (13.14 ± 19.96) groups declined *(*p<0.001), however after controlling the initial severity of nausea, the declining extent was more noticeable in the intervention group than in the placebo. The episodes of nausea (37.1% vs 65.7%, p=0.006), and retching (20% vs 45.7%, p=0.028) were significantly lower in the intervention group than in the placebo group. Administration of antiemetic medications was lower in the intervention than in the placebo group (37.1% vs 65.7%, p=0.009)
Cheshmeh et al., 2021 [21]	Obese women with PCOS	Both the test group and placebo group were administered a low-calorie diet. The test group also consumed 3 g/day of green cardamom and the placebo group had 3 g of starch powder	Double-blind randomized controlled trial	194	None	Anthropometric indices were improved in both studied groups (p<0.001). LH, androstenedione, and DHEA decreased (p<0.001), and FSH increased (p<0.001) in the test group. TNF-α, IL-6, and CRP serum levels decreased in the test group (p<0.001). TNF-α and CRP gene expression decreased in the test group (p<0.001)
Cheshmeh et al., 2021 [22]	Obese women with PCOS	Both the test group and placebo group were administered a low-calorie diet. The test group also consumed 3 g/day of green cardamom and the placebo group had 3 g of starch powder	Double-blind randomized controlled trial	194	None	Anthropometric indices were decreased after intervention in both groups. Glycemic indices and androgenic hormones were significantly improved in the test group. The expression levels of FTO, CPT1A, LEPR, and LAMIN were down-regulated (p<0.001), and PPAR-y was upregulated in the test group after intervention with green cardamom (p<0.001)

Turmeric (*Curcuma longa*)

Turmeric, a gold-colored spice derived from the *Curcuma longa* plant, has been a staple in traditional medicine for centuries. With its active compound, curcumin, turmeric has anti-inflammatory, antioxidant, anti-microbial, antiangiogenic, anti-mutagenic, wound healing, and pro-apoptotic properties [24]. It can also act synergistically with other plant polyphenols such as resveratrol, catechins, piperine, quercetin, and genistein [24]. Throughout history, this spice has been used in traditional Asian medicine and Ayurveda to treat digestive disorders, rheumatoid arthritis, conjunctivitis, liver diseases, urinary tract infections, smallpox, chickenpox wounds, and menstrual irregularities [25]. While reviews on the applications of turmeric’s anti-inflammatory and antioxidant effects in conditions such as autoimmune conditions, cardiovascular disease, cancers, and diabetes have been thorough, its impact on female reproductive health is a topic that has not gained as much attention in literature reviews. The most common reproductive conditions that have been studied for the effects of turmeric through experimental research and clinical trials are PCOS, endometriosis, and dysmenorrhea. 

Endometriosis is a medical condition characterized by the presence of the endometrial gland and stroma tissue growing outside of the uterus, causing pain and inflammation as the menstrual cycle progresses [26]. In a study by Cao et al. [26] using the MTT (3-(4, 5-dimethylthiazol-2)-2, 5-diphenyltetrazolium bromide) assay and Hematoxylin and Eosin staining, curcumin decreased human ectopic and eutopic stromal cell growth. Vascular endothelial growth factor (VEGF) expression also decreased with curcumin treatment. Findings suggested that curcumin reduces endometriotic cell survival as indicated by an increased percentage of G1-phase cells, and a decreased percentage of S-phase cells [26]. Given the experimental study design and small sample size, the applicability of these findings to larger animal and human populations needed further investigation. This is seen in a study by Swarnakar and Paul [27] where matrix metalloproteinase (MMP)-9 activity increased with the severity of endometriosis, and curcumin was shown to reverse this increase in activity to near control levels. This decreased MMP-9 activity was associated with decreased expression of tumor necrosis factor-α (TNF-α), highlighting the anti-inflammatory properties of curcumin [27]. However, when assessing human populations in a clinical trial by Gudarzi et al. [28], women with endometriosis reported no significant changes with curcumin in pain and quality of life through the Endometriosis Health Profile questionnaires. Perhaps statistical significance may be achieved with a greater sample size, as this study's sample size was only 34. With the design of this study involving surveys and questionnaires, bias is another potential factor to consider in the outcome. 

Another reproductive condition studied for the therapeutic benefits of turmeric is the hormonal disorder PCOS. In a letrozole-induced PCOS mouse model, Zahoor ul Haq Shah and Shrivastava [29] found that while letrozole caused an increase in LH and a decrease in estrogen, progesterone, FSH, and adiponectin, turmeric extract administration reversed these changes. However, the study had a very low sample size and may not be generalizable. In a randomized controlled trial on women with PCOS by Shorevardi et al. [30], the effects of metformin alone versus metformin in combination with curcumin nanomicelle on blood triglycerides, high-density lipoprotein cholesterol (HDL-C), low-density lipoprotein cholesterol (LDL-C), total cholesterol, plasma glucose, alanine aminotransferase (ALT), and aspartate aminotransferase (AST), insulin resistance (HOMA-IR), and insulin-sensitivity check index (QUICKI) were assessed. The curcumin group had significantly lower fasting insulin, HOMA-IR, LDL-C, total cholesterol, and triglycerides, and higher HDL-C than metformin alone, suggesting a synergistic effect of the two compounds [30]. This study did not administer a placebo for curcumin in the metformin-only group, which could lead to biases that create confounding factors in these findings. Follow-up studies should also evaluate the effects of cardamom alone without Metformin, to further evaluate if a synergistic versus additive effect is present with the two compounds. In another study, Asan et al. [31] found a decrease in body weight, body fat, waist circumference, fasting blood glucose levels, fasting insulin levels, HOMA-IR, and CRP in their curcumin treatment group compared to placebo in eight weeks. However, unlike the previous study, they did not find any significant differences in total cholesterol, LDL-C, HDL-C, and triglyceride levels [31]. This may be due to the study duration being only eight weeks, which is significantly shorter than the previous three-month study. More extensive clinical trials with well-thought-out methodology are needed for the further investigation of these findings and how curcumin would compare to the current first-line treatments for PCOS.

Curcumin has also shown promise in treating primary dysmenorrhea. Dyawapur et al. [32] found a significant decrease in post-test versus pre-test reports of pain in young adult females with dysmenorrhea who were treated with turmeric water as well as with cinnamon tea. Both cinnamon tea and turmeric water showed equal efficacy in reducing pain levels [32]. Potential bias is introduced in this study design as the participating women are required to provide pre-test and post-test descriptions of their pain levels with treatment. In a prospective case-control study by Okuyan et al. [33], the authors assigned women with diagnosed primary dysmenorrhea to two groups. Both groups received Naproxen while group 2 had turmeric powder added to their treatment regime during menstruation. While both groups reported a significant decrease in pain levels with their respective treatments, the decrease of pain in group 2 was significantly greater than that of group 1 with naproxen alone [33]. Placebo was not given to group 1 in place of turmeric powder and could have influenced study outcomes. Further studies of dysmenorrhea treatment with turmeric alone without naproxen can provide further information on its therapeutic potential as an alternative to pharmacologic therapies. While these studies show promising results in the treatment of primary dysmenorrhea with turmeric supplementation, a randomized controlled trial by Bahrami et al. [34] where premenstrual syndrome (PMS) and dysmenorrhea were evaluated using a premenstrual syndrome screening tool (PSST) and the visual analog scale (VAS), curcumin had no significant difference in alleviating pain compared to placebo. Further studies with larger sample sizes may provide more reliable and reproducible insights.

In the toxicity studies of turmeric and curcumin, a review by Soleiman et al. [35] concludes that turmeric and curcumin are nontoxic for humans with oral administration. They are also non-mutagenic and safe in pregnancy in animals, while studies in humans are insufficient. However, Tossetta et al. [36] discuss the various studies that show contrasting data on curcumin intake during early pregnancy in animal models with possible negative consequences on the blastocyst stage, oocyte maturation, oocyte fertilization, and embryonic development. These factors should be taken into consideration, and further research is necessary in this regard.

The results and characteristics of the selected cases are outlined in Table 3.

**Table 3:** Characteristics of the research papers on the applications of turmeric in female reproductive health

Research studies						
Reference	Subjects	Treatment	Study design	Sample size (n)	Adverse effects	Main results
Cao et al., 2017 [26]	For ectopic endometriotic stromal cells, patients aged 24-45 years with endometriosis, for eutopic endometrial stromal cells, premenopausal patients who had undergone hysteroscopy for infertility	Various concentrations (0, 20, and 50 µmol/L) of curcumin were added to endometrial stromal cell cultures for 48 hours	Experimental study	14 ectopic and 9 eutopic	None	Curcumin decreased human ectopic and eutopic stromal cell growth (p<0.05). Vascular endothelial growth factor (VEGF) expression also decreased with curcumin treatment (p<0.05). Curcumin reduces endometriotic cell survival as indicated by an increased percentage of G1-phase cells, and a decreased percentage of S-phase cells (p<0.05)
Swarnakar and Paul, 2009 [27]	Female Balb/c mice 6-8 weeks old	48 mg/kg body weight of curcumin as well as vehicle administered intraperitoneally, once daily for 10 days (for 5 mice) and 20 days (for 5 mice) from day 15 post-endometriosis	Experimental study	40	None	MMP-9 activity increased gradually in endometriotic tissues with severity and curcumin treatment reversed the MMP-9 activity near to control value (p<0.001). Curcumin administered either post- or pre-endometriosis arrested endometriosis in a dose-dependent manner (p<0.001). MMP-9 activity (p<0.001) and expression, as well as TNF-α expression, decreased with curcumin exposure. Curcumin prevented lipid peroxidation and protein oxidation in endometrial tissues (p<0.001)
Gudarzi et al., 2024 [28]	Women with endometriosis	Curcumin capsules with a dose of 500 mg were given to the intervention group twice a day for 8 weeks, and the placebo with the same dose was given to the control group	Triple-blind randomized controlled trial	34	None	No statistically significant difference in the amounts of usual pain (p=0.496) and pain at its worst (p=0.320), quality of life (p=0.556), and visual pain (p=0.845) between curcumin and control groups
Zahoor ul Haq Shah and Shrivastava, 2022 [29]	Female Swiss Albino mice with letrozole-induced PCOS	PCOS untreated, PCOS + Turmeric Extract (175 mg/kg), or PCOS + Metformin (150 mg/kg). Turmeric Extract and Metformin were combined in a 0.9% NaCl solution and taken orally for 30 days	Experimental study	6	None	Letrozole-induced PCOS caused an increase in LH (P=0.001), glucose, cholesterol, and triglycerides, and a decrease in estrogen, progesterone, FSH, and adiponectin (p=0.0001). Turmeric extract reversed the levels of LH, glucose, cholesterol, triglycerides, estrogen, progesterone, FSH (p=0.0001), and adiponectin (p=0.01)
Shorevardi et al., 2021 [30]	Women with PCOS, diagnosed according to the Rotterdam criteria	Group 1 received 500 mg metformin three times daily and group 2 received 80 mg/day capsule of curcumin nanomicelle and 500 mg metformin three times a day for 3 months	Randomized controlled trial	50	None	After treatment, fasting insulin, HOMA-IR, and total testosterone in group 2 were significantly lower than those in group 1 (p<0.05). LDL-C levels in groups 1 and 2 were 117.9 ± 24 and 91.12 ± 19.46 mg/dL, respectively (p<0.01). HDL-C levels were increased with curcumin (p<0.05). Total cholesterol decreased with curcumin (p<0.05), and triglycerides decreased with curcumin (p<0.01)
Asan et al., 2020 [31]	Women aged 20-35 with a BMI between 25-35 kg/m^2^ who were newly diagnosed with PCOS based on the Rotterdam criteria	A highly bioavailable formulation of curcumin was administered at a daily dose of 93.34 mg (2 capsules) for 8 weeks	Single-blind randomized controlled trial	15	None	Body weight, body fat mass, and waist circumference were lower in the curcumin group than in the placebo group (p<0.05). Significant differences in fasting blood glucose levels, fasting insulin levels, HOMA-IR, and CRP levels in the curcumin group compared to the placebo (p<0.05). No significant differences in total cholesterol, LDL-C, HDL-C triglycerides, and hormone levels between both groups (p>0.05)
Dyawapur et al., 2018 [32]	Degree girls staying at hostels in Vijayapur, India	30 mL of turmeric water administered directly after the onset of menstruation and preferably after meals at 0 hours, 8 hours, and 16 hours. Pain level was assessed after 20 minutes of administration of turmeric water	Pre-test and post-test design	60	None	A significant difference between the pre-test and post-test effect of cinnamon tea (t28=15.78, df=28) and turmeric water (t28=2.11, df28=2.05) in reducing the intensity of pain from moderate pain to mild pain. No significant difference between cinnamon tea and turmeric water scores (t28=0.5, df=2.05)
Okuyan et al., 2021 [33]	Nulliparous women between 15-45 years, BMI <25 kg/m^3^, with a diagnosis of primary dysmenorrhea	Naproxen (750 mg/day) was given to all patients in Group 1 and Group 2, and 1 g/day of turmeric powder was added to the treatment of Group 2 patients to be consumed during menstrual bleeding	Prospective case-control study	75	None	The median pain score of the population decreased from 9.17 to 2.81 after the treatment, with a Z-score of -10.64. The decrease in VAS scores was significant in both groups (p=0.001 for both). The percentage of VAS score decrease (61.7% vs 76.8%) and the absolute score decrease (5.6 vs 7.0) were significantly higher in Group 2 compared to Group 1 (p=0.001 for both)
Bahrami et al., 2021 [34]	Young women with diagnoses of both PMS and dysmenorrhea	Each subject received one capsule (500 mg of curcuminoid, or placebo) daily, from 7 days pre- until 3 days post-menstruation for three successive menstrual cycles	Triple-blind randomized controlled trial	62	None	PSST scores were significantly lower in both the curcumin (32.5 ± 9.8 vs. 21.6 ± 9.8); and placebo groups (31.7 ± 9.4 vs. 23.4 ± 12.8). There was a significant reduction in dysmenorrhea pain in both the curcumin and placebo groups (64% and 53.3%, respectively)

Tulsi (*Ocimum tenuiflorum*)

Tulsi or holy basil is a fragrant herb in the *Lamiaceae* family, native to the Indian subcontinent [37]. With a history of over 3000 years in Ayurvedic medicine, Tulsi is revered for its culinary and medicinal properties and is referred to as the “Medicine of Life” in Ayurveda [37]. With its antispasmodic, appetite stimulative, carminative, galactagogue, antioxidant, and anti-inflammatory properties, Tulsi has been employed for various digestive issues such as stomach cramps, gastroenteritis, vomiting, and constipation, and upper respiratory conditions such as whooping cough [37,38]. The phytochemical constituents of Tulsi plants are eugenol, carvacrol, sesquiterpene hydrocarbon caryophyllene, phenolics, flavonoids, terpenoids, fatty acids, mucilage, polysaccharides, linoleic acid, and sitosterol [38]. While not many experimental studies have been done, in this report, we focus on the effects of Tulsi on female reproductive health such as fertility, PCOS, and reproductive hormones.

In a 2019 study done by Poli and Challa [39], the antifertility effects of eugenol, one of the potent bioactive ingredients in Tulsi, and Tulsi leaf extract were studied in female albino rats. Healthy rats were administered eugenol and Tulsi leaf extract orally for 15 days and the study found a prolongation of the estrous cycle with eugenol administration and no significant effect with Tulsi leaf extract. While eugenol elevated both estrogen and progesterone, Tulsi extracts only elevated progesterone levels. Both compounds caused elevations of ovarian proteins. With the prolongation of the estrous cycle and consequent reduction in cycle frequency, the authors suggest that ovulation would be reduced, and fertility impaired with eugenol exposure. However, as mentioned, although eugenol is present in Tulsi leaf extract, Tulsi leaf extract alone did not produce any changes in estrous cycle duration, suggesting possible confounding factors in the study that require further investigation [39]. The study also had a significantly small sample size, questioning the validity of these findings. A follow-up study by Poli and Reddy [40], showed a significant reduction in the number of corpora lutea of pregnancy and the number of live fetuses in rats exposed to eugenol and in rats exposed to Tulsi leaf extract, compared to the saline control. There was also a decrease in placental and fetal weights within the two test groups. The anti-implantation activity of the eugenol and Tulsi extract groups was calculated to be 87.17% and 79.48% respectively. The antifertility activities were calculated to be 83.33% in both test groups [40]. While these studies provide some preliminary evidence for the antifertility effects of Tulsi and open the doors for further exploration of its use as a contraceptive, the findings should be reproduced using larger sample sizes for validity and generalizability. 

Tulsi plant extract also shows some efficacy in the treatment of PCOS in a study of female Wistar rats with letrozole-induced PCOS by Farhana et al. [41]. Here, the authors administered letrozole orally in the rats for 21 days for the induction of PCOS. 100 mg/kg and 200 mg/kg doses of Tulsi extract were then administered using carboxymethyl cellulose (CMC) as a vehicle. While the letrozole caused changes in serum sex hormone levels, glucose, and antioxidant activity that are congruent with PCOS, Tulsi extract was able to reverse these changes back to their baseline levels. Treatment with Tulsi extract also led to the disappearance of subcapsular cysts that formed in letrozole-treated rats and decreased the incidence of pyknotic granulosa cells. The effects of the leaf extract were comparable to the gold standard clomiphene citrate which is used for ovulation induction in patients with PCOS [41]. Clinical trials with sufficient sample sizes are needed to determine how these effects translate into human populations.

Apart from the reproductive toxicity of Tulsi, a study of healthy Wistar rats showed that oral administration of Tulsi extract was not toxic to clinical, hematological, biochemical, and histopathological parameters of male and female rats at doses of up to 1000 mg/kg/day over 28 days [42]. Human studies on the toxicity profile of the herb are limited.

The selected studies and their findings are presented in Table 4.

**Table 4:** Characteristics of the research papers on the applications of Tulsi in female reproductive health

Animal studies						
Reference	Subjects	Treatment	Study design	Sample size (n)	Adverse effects	Main results
Poli and Challa, 2019 [39]	Healthy 4-month-old female Wistar albino rats	Group 1 control rats were administered 1 mL of saline. Group 2 rats were intramuscularly administered 0.4 mL/day of pure compound eugenol (99%) for 15 days. Group 3 rats orally administered 500 mg/kg body weight/day of Tulsi leaf extract for 15 days	Experimental study	6	None	The Estrous cycle was prolonged with eugenol and no significant changes with Tulsi leaf extract administration (p<0.001). Eugenol elevated serum estradiol and progesterone levels but Tulsi leaf extract elevated only progesterone (p<0.001). Elevated ovarian proteins were seen in both test groups (p<0.001)
Poli and Reddy, 2020 [40]	Healthy adult female Wistar albino rats	Group 1 control rats were administered 1 mL of saline. Group 2 rats were intramuscularly administered 0.4 mL/day of pure compound eugenol (99%) for 20 days. Group 3 rats orally administered 500 mg/kg body weight/day of Tulsi leaf extract for 20 days	Experimental study	6	None	Group 3 rats showed a reduction (p<0.05) in the number of corpora lutea of pregnancy and the number of live fetuses. Fetal and placental weights were also significantly (p<0.05) decreased in the Eugenol and Tulsi extract groups compared to the control. Anti-implantation activity was 87.17% and 79.48% in the Eugenol and Tulsi extract groups. The antifertility activity was 83.33% in both test groups
Farhana et al., 2018 [41]	Adult female Wistar albino rats with letrozole-induced PCOS	Group 1 control, Group 2 PCOS untreated, Group 3 standard administered Clomiphene citrate 1 mg/kg in 0.5% CMC per oral, Group 4 and 5 administered Tulsi extract 100 mg/kg and 200 mg/kg body weight respectively in 0.5% CMC per oral for 15 days	Experimental study	6	None	Letrozole increased testosterone, triglycerides, total cholesterol, LDL-C, and glucose (p<0.001), and decreased HDL-C estradiol and progesterone (p<0.001). Groups 3, 4, and 5 had a decrease in testosterone and glucose (P<0.001), and an increase in estradiol (p<0.001 for Group 3 and 5, P<0.01 for Group 4). All treatment groups decreased triglycerides (p<0.001), total cholesterol (p<0.001), and LDL-C (p<0.01 for Group 4, p<0.001 for Group 3 and 5), and increased HDL-C (p<0.01 for Group 4, p<0.001 for Group 5). Letrozole depleted antioxidants (p<0.001), and Tulsi extract increased their levels (p<0.001). Cysts disappeared with Tulsi extract treatments

Ginger (*Zingiber officinale*)

Ginger (*Zingiber officinale*) is a well-known antioxidant recognized for its medicinal properties and has been a part of traditional medicine and Ayurveda for centuries [43]. Its volatile components such as monoterpenoid hydrocarbons and sesquiterpenes give ginger its characteristic taste and smell, while its nonvolatile bioactive components such as gingerols, shogaols, paradols, and zingerone give ginger its antioxidant properties by inhibiting xanthine oxidase, which is involved in the generation of reactive oxygen species (ROS) [43,44]. ROS plays important roles in modulating female reproductive processes including oocyte maturation, follicular atresia, ovulation, fertilization, and luteal regression [43]. Through its effects on ROS, ginger shows promise as an intervention that could contribute to maintaining reproductive health and supporting physiological pathways that are crucial for female reproduction. Ginger has also been extensively studied for treating nausea and vomiting associated with early pregnancy.

Clomiphene citrate, today’s standard for the treatment of infertility and PCOS comes with a host of psychological side effects such as bloating, mood swings, and depressed mood [45]. In animal studies, Yilmaz et al. [46] investigated the effects of ginger powder on ovarian folliculogenesis and implantation in rats. The authors administered varying doses of ginger powder or distilled water control to rats through either five days (one estrous cycle) or 10 days (two estrous cycles). They subsequently discovered a higher antral follicle count and ovarian stromal VEGF in the 10-day low-dose ginger treatment group, with no significant changes in the high-dose treatment group compared to the control. Ginger appears to enhance long-term implantation in rats at shorter doses and could have potential as a natural alternative or adjunct with minimal side effects in boosting fertility [46]. This is further supported in a study by Usman et al. [47] where ginger honey supplementation in stress-induced mice increased estrogen and glutathione levels significantly, while not affecting cortisol levels in comparison to standard feed alone. However, the coadministration of honey in this study serves as a confounding variable. Further, no control was used for ginger honey in the group that received standard feed alone.

Atashpour et al. [48] also explored the effects of ginger extract on sex hormones in PCOS-induced rats compared to the gold standard clomiphene citrate. They discovered that ginger at higher doses was effective in reversing the changes in estrogen, progesterone, and FSH that were caused by PCOS induction [48]. High-dose ginger was suggested to be an effective alternative therapy with no side effects for the management of PCOS. Ginger in the management of PCOS has also suggested efficacy in human studies. In women with PCOS, one study by Bonab [49] found that 12 weeks of ginger supplementation and Pilates exercise were able to significantly reduce LH, testosterone, and insulin levels. They were also both equally capable of increasing FSH and sex hormone-binding globulin (SHBG). While the combination of both therapies together had the greatest positive effect, both ginger and Pilates training alone are also beneficial therapeutic options for improving PCOS [49]. The pre-test and post-test study design used in this study may introduce biases that influence the study outcome and undermine its reliability. All these studies discussed so far on ginger also had very low sample sizes, and further investigation is needed.

In the United States, ovarian cancer is the most lethal gynecologic malignancy and poses significant challenges in prevention and treatment [50]. Current theories suggest that incessant ovulation and excess gonadotropin secretion may contribute to ovarian carcinogenesis through a pro-inflammatory state. In the pursuit of effective treatment strategies, a study by Rhode et al. [50] explores ginger due to its antioxidant and anticarcinogenic properties. Specifically, the ginger component 6-shogaol demonstrated remarkable growth inhibition in epithelial ovarian cancer cells, highlighting its potential as a therapeutic agent. Further, ginger treatment led to the inhibition of Nuclear factor kappa-light-chain-enhancer of activated B cells (NF-κB) activation and reduced secretion of angiogenic factors, suggesting that dietary agents like ginger could hold promise in both the treatment and prevention of ovarian cancer [50]. The applicability of these findings to animal and human subjects needs further evaluation. 

In the management of dysmenorrhea, ginger has undergone some human studies as well. A randomized controlled trial by Naveed et al. [51] compared the effects of ginger and vitamin E on pain levels via the VAS scale and quality of life questionnaire in females with dysmenorrhea. Ginger tea and vitamin E were both effective in reducing dysmenorrhea symptoms, pain, and VAS scale levels. Placebo groups also showed improvement in these parameters, suggesting a psychological aspect to the treatment of dysmenorrhea as well [51]. The additional components involved in the making of ginger tea also pose confounding variables in the study. In a pre-test post-test style study done by Harjai and Chand [52], the percentage of participants who reported severe pain before ginger application compared to after ginger application was significantly reduced. However, this pre-test and post-test study design again introduces potential biases or threats to validity and limits the applicability of the study. However, given that ginger has no adverse effects as found in a meta-analysis by Kim et al. [53], it’s a noteworthy alternative warranting further investigation in primary dysmenorrhea management. 

Many pharmaceuticals have proven to have side effects on fetuses when consumed by pregnant mothers. Natural herbal remedies for common conditions experienced during pregnancy are hence, one of the most important applications of Ayurvedic and alternative medicine. Several studies have investigated ginger’s potential as an antiemetic in the treatment of pregnancy-associated nausea and vomiting. Viljoen et al. [54] conducted a meta-analysis and systematic review to ascertain the effectiveness of ginger in treating nausea and vomiting and assess its safety in pregnancy. While ginger did not significantly affect vomiting episodes, it did show some promise in reducing nausea associated with pregnancy [54]. This is further supported in another meta-analysis by Thomson et al. [55]. With its lack of notable side effects and adverse events, ginger may be an effective option for pregnant women. The selected cases discussed here are presented alongside their findings in Table 5.

**Table 5:** Characteristics of the research papers on the applications of ginger in female reproductive health

Research studies						
Reference	Subjects	Treatment	Study design	Sample size (n)	Adverse effects	Main results
Yilmaz et al., 2021 [46]	Female albino rats in estrous cycle	100 mg ginger powder, 200 mg ginger powder, or 2 cc distilled water (control group) was given to 3 subgroups. Ginger powder was mixed with 2 cc distilled water and administered by gavage. The control group also received 2 cc of distilled water by gavage	Experimental study	7	None	In the 5-day treatment group, antral follicle count and ovarian stromal VEGF were significantly high in the 100 mg ginger subgroup in comparison to the control group (p<0.05). In the 10-day treatment group, endometrial VEGF and ovarian stromal eNOS were significantly high in the 100 mg ginger subgroup in comparison to the control group (p<0.05). There was no statistically significant difference at 200 mg ginger dose both in 5-day and 10-day treatment groups
Usman et al., 2021 [47]	Female Balb/c mice aged 2-3 months	Group 1 as a control group) is given standard feed, and group 2 as ginger honey as much as 42 mg/20 g body weight is given by oral sonde and standard feed for 14 days	Experimental study	5	None	42 mg/20 g BW of ginger honey administration for 14 days increased 1.892 ng/dl of cortisol (p=0.165), increased 2.438 ng/dl of glutathione (p=0.002), and also increased 22.754 ng/ml estrogen levels in induced stress Balb/c female mice (p=0.001)
Atashpour et al., 2017 [48]	Adult female Wistar rats age 7-8 weeks	The control group (no intervention for 60 and 89 days), sham group (given distilled water and ethyl alcohol intraperitoneally daily for 60 and 89 days), and 7 experimental groups given estradiol valerate (PCOS inducing agent, intramuscular) alone and with 100 mg/kg clomiphene or different doses of ginger extract (175 and 350 mg/kg) orally daily for 60 and 89 days	Experimental study	7	None	PCOS-induced group LH and estrogen concentration increased while progesterone and FSH concentration decreased (p<0.05) compared to the control group. In clomiphene and ginger extract groups, there was an improvement in hormonal secretion (p<0.05). Clomiphene had a better effect on improving sexual hormones in PCOS than lower-dose ginger. Ginger extract at higher doses has better effects in improving PCOS
Bonab, 2020 [49]	Women aged 20-25 with PCOS	4 groups including control, Pilates exercise, combined (Exercise + Ginger supplement), and Ginger supplementation. The exercise intervention groups performed Pilates exercises for 12 weeks and the supplementation group consumed 1 g of ginger capsules three times per day	Quasi-experimental study with pre-test and post-test design	10	None	LH (p<0.05), testosterone (p<0.05), and insulin (p<0.05) levels decreased in the intervention groups compared to the control group. The FSH (p<0.05) and SHBG (p<0.05) indices in the intervention groups were higher than the control. The findings indicated the effects of treatments on the indices studied (p<0.05). While the combined treatment had the greatest effect, there were no significant differences between the exercise group and ginger supplementation group (p>0.05), except in the weight index the superiority was observed with the exercise group (p<0.05)
Rhode et al., 2007 [50]	SKOV3 ovarian cancer cells were obtained from the American Type Culture Collection. SKOV3, CaOV3, and ES-2 cells were originally harvested from patients with recurrent ovarian cancer	Activation of NF-κB and production of VEGF and IL-8 was determined in the presence or absence of ginger (75 µg/mL)	Experimental study	N/A	None	Ginger treatment of cultured ovarian cancer cells induced profound growth inhibition in all cell lines tested (p<0.05). We found that in vitro, 6-shogaol is the most active of the individual ginger components tested (p<0.05). Ginger treatment resulted in the inhibition of NF-kB activation as well as diminished secretion of VEGF and IL-8 (p<0.05)
Naveed et al., 2022 [51]	Females of reproductive age having dysmenorrhea for more than one year	Group 1 received 500 mg ginger 1 cup tea/day (200 mL). Group 2 received Vitamin E 100 IU 1 tablet/day. Group 3 control starch tablets 250 mg one tablet/day	Randomized controlled trial	30	None	There is a significant correlation in the results of the VAS scale throughout the study. The level of significance indicates that the VAS scale of pain showed various results in the pain levels of patients having ginger tea and vitamin E capsules for the pain management of menstruation of females. There is also a significance noticed in the placebo group (p<0.01 or p<0.05 throughout)
Harjai and Chand, 2018 [52]	Adolescent female nursing students	Experimental group 1 was given ginger two days before the onset of menses and continued through the first three days of menses. Experimental group 2 was given ginger only for the first three days of menses	Experimental study with pre-test and post-test design	30	None	Ginger application has a significant effect in reducing pain in both group 1 and group 2. Group 2 had an even more effective reduction in pain level than Group 1 (p<0.05)
Viljoen et al., 2014 [54]	Randomized controlled trials involving human participants and investigating ginger for the treatment of nausea and vomiting in pregnancy	Any form of orally administered ginger intervention (fresh root, dried root, powder, tablets, capsules, liquid extract, and tea) compared with an inert (placebo) or active ingredient	Meta-analysis	12	Allergic reaction, arrhythmia, dehydration, spontaneous abortion, belching, burning sensation, diarrhea, dry retching or vomiting, headaches, drowsiness, and heartburn	Ginger significantly improved the symptoms of nausea when compared to placebo (p=0.0002). Ginger did not significantly reduce the number of vomiting episodes compared to placebo, although there was a trend towards improvement (p=0.06). Subgroup analyses favor the lower daily dosage of <1500 mg ginger for nausea relief. Ginger did not pose a significant risk for spontaneous abortion compared to placebo (p=0.15), or to Vitamin B_6_ (p=0.19). Similarly, ginger did not pose a significant risk for the side effects of heartburn or drowsiness
Thomson et al., 2014 [55]	Placebo-controlled trials with a satisfactory score on the Cochrane Risk of Bias assessment tool	One study used 5 biscuits per day, each containing 500 mg of ginger, whereas the others used either capsules or syrup containing approximately 1 g of ginger daily	Meta-analysis	6	None	The use of ginger for at least 4 days is associated with a 5-fold likelihood of improvement in nausea and vomiting in early pregnancy (p<0.0001)

Ashwagandha (*Withania somnifera*)

Ashwagandha (*Withania somnifera*), also known as Indian ginseng or winter cherry, is a herbaceous plant belonging to the Solanaceae family [56]. Originating from the arid regions of India, the herb's name reflects its horse-like aroma, symbolizing the strength and vigor it is believed to impart. Today the herb has become quite popular and is farmed across South and Central Asia as well as Africa [57].

Regarded as a botanical marvel, ashwagandha is esteemed for its adaptogenic properties that contribute to vitality, resistance, and various health-promoting benefits. This can be attributed to its diverse array of bioactive compounds. In examining the ashwagandha plant, over 50 chemical constituents have been identified, with major components comprising steroidal alkaloids and lactones collectively referred to as withanolides [58]. Acknowledged for its adaptogenic efficacy, ashwagandha has not only found applications in traditional medicine systems globally but has also maintained a significant role as a fundamental element in Ayurvedic practices. This adaptogenic nature is intricately connected to its potential to modulate the body's stress response, enhance resilience, and promote homeostasis. Beyond stress management, the herb has been associated with cognitive function, anti-inflammatory effects, immune system support, and potential benefits in reproductive health [58].

A study by Rahi et al. [59] looked at the effects of herbal garlic extract and ashwagandha in comparison to the antibiotic ciprofloxacin in the treatment of endometritis and repeat breeding conditions in crossbred cows. At the subsequent estrous cycle following treatment, the majority of cows treated with either of the herbs or the antibiotic showed improvement of endometritis as evidenced by clear mucus discharge, negative reaction to the white slide test, and reduction in bacterial load in the cervical mucus. The group of cows treated with combined ashwagandha and garlic showed better conception rates that were comparable to that of the ciprofloxacin treatment group. The authors concluded that endometritis could be treated effectively with the combination of ashwagandha and garlic, owing to their synergistic effects in combating infection and immunomodulation, and can replace the need for antibiotic treatments [59]. The very small sample size used in the study and experimental design necessitate further research into how this would translate to human populations before we can consider promoting these herbs in place of first-line pharmacotherapies. 

The perimenopausal period in women is marked by many hormonal and physiologic changes that manifest as climacteric symptoms such as hot flashes, sleep issues, and vaginal dryness [60]. Gopal et al. [60] performed a randomized controlled study referencing ashwagandha's role on climacteric symptoms in women during the perimenopausal period. Women with climacteric symptoms were randomly allocated and treated with ashwagandha root extract or placebo and outcomes were measured using the menopause rating scale (MRS), menopause-specific QoL (MENQoL), hot flash score, and changes in estradiol, FSH, LH, and testosterone. The authors found significant reductions in these parameters with ashwagandha supplementation compared to the control, suggesting that the herb can be a safe and effective treatment option for perimenopausal symptoms [60]. It should be noted, however, that potential biases may be present when reporting subjective symptoms on these questionnaires such as hot flashes, anxiety, memory, etc. 

Endometrial cancer, a malignancy originating in the lining of the uterus (endometrium), represents a significant health concern. Its prevalence has been steadily rising, making it one of the most diagnosed gynecological cancers worldwide. The incidence of endometrial cancer is particularly notable in postmenopausal women, although cases can also occur in premenopausal individuals. While several studies have looked at the anticancer properties of ashwagandha, Xu et al. [61], found in vitro, ashwagandha has been shown to inhibit the proliferation of human endometrial cancer cells. It does so through Withaferin A, a naturally occurring compound within the ashwagandha plant. Withaferin A exhibits an inhibitory effect on TGF-β through the modulation of TGF-β signaling and the inhibition of TGF-β dependent Smad2 phosphorylation. While this study references specifically endometrial cancers, more research is required to know if this is a viable treatment option extendable to cervical or ovarian cancers [61]. Another issue facing women is sexual functioning, which is often influenced by a range of psychologic, pathophysiologic, and interpersonal relationship factors [62]. In a randomized controlled trial by Dongre [62], the potential of ashwagandha to improve female sexual function as measured by the Female Sexual Function Index (FSFI) Questionnaire and the Female Sexual Distress Scale (FSDS) was evaluated. Ashwagandha showed significant potential in improving sexual function in the areas of desire, arousal, lubrication, orgasm, satisfaction, pain, and FSFI and FSDS scores in healthy women over eight weeks [62]. Given the smaller sample size and the short duration of this study, more rigorous clinical trials may be needed to further assess the role of this herb in managing sexual dysfunction. The employment of questionnaires in the study design again introduces the potential for bias in the scores and results. However, research seems to be heading in the right direction as there have been no adverse effects noted in both male and female volunteers who consumed ashwagandha for eight weeks in a study by Verma et al. [63]. 

Table 6 presents the findings of the selected research papers in this section.

**Table 6:** Characteristics of the research papers on the applications of ashwagandha in female reproductive health

Research studies						
Reference	Subjects	Treatment	Study design	Sample size (N)	Adverse effects	Main results
Rahi et al., 2013 [59]	Repeat breeding crossbred cows	Eight groups (A-H) treated with phosphate-buffered saline (PBS) once a day (OD) intrauterine (I/U), ciprofloxacin OD I/U, 30 mL garlic extract OD I/U, 30 mL ashwagandha extract OD I/U, 15 g ashwagandha powder twice daily orally, 30 mL garlic OD I/U + 15 g ashwagandha powder twice daily orally, 30 mL garlic extract with 30 mL ashwagandha extract OD I/U, respectively at estrus	Experimental study	8	None	A decline in pH in all groups after treatment (p<0.05). A significant decrease (p<0.05) in the pH of groups B-H was observed compared to group A. Decreased pH of group G and H compared to group D, and decreased pH of group G compared to group E. There was a significant decline (p<0.05) in bacterial load in the cervical mucus of all the groups except group A. The percent reduction was highest in group B followed by group C, F, D, G, H, E, and A cows. Bacterial count in groups B-H at subsequent estrus after treatment varied significantly (p<0.01) as compared to group A
Gopal et al., 2021 [60]	Women with climacteric symptoms	Placebo or 300 mg of an ashwagandha root extract twice daily	Randomized, double-blind controlled study	50	None	Ashwagandha was associated with a reduction in MRS score (p<0.0001), reflected by significant reductions in psychological (P=0.0003), somato-visceral (p=0.0152), and urogenital (p<0.0001) domains. Ashwagandha intake reduced MENQoL scores (p<0.0001), increased serum estradiol (p<0.0001), and reduced FSH (p<0.0001) and LH (P<0.05) compared to placebo. No difference in testosterone levels between groups
Xu et al., 2021 [61]	Cancerous KLE endometrial cells and normal, healthy THESCs endometrial cells	Viability assay: cells were exposed to different withaferin A concentrations viz 0, 2.5, 5, 10, 20, 40, 80, and 180 µM for 48 h at 37 °C. Post-treatment, 0.5% MTT reagent (Sigma) was supplied to each well and incubation was performed at 37 °C for an additional 4 hours. Proliferation Assay: KLE cells (2 × 10^5^ cells/well) were treated with 10 µM concentration of withaferin A within 96-well plates maintaining EdU (5-ethynyl-2’-deoxyuridine) medium for 48 hours	Experimental study	2 × 10^5^ cells/well in a 96-well plate	None	THESCs cells post-48 hours prolonged withaferin A treatment (0–160 µM) were observed to be decreased from 100% to almost 25% with an IC50 value of 76 µM (p<0.05). Endometrial cancer KLE cells, withaferin A showed significant inhibition of KLE cell proliferation with an IC50 value of 10 µM (p<0.05)
Dongre et al., 2015 [62]	Women aged 21-50 who were in a steady heterosexual relationship for over a year, previously engaged in sexual function for several years	High-concentration ashwagandha root extract (HCARE) was given in the dose of one capsule of 300 mg twice per day orally after food with a glass of plain water, over 8 weeks. The same protocol was followed for the placebo	Randomized double-blind controlled trial	25	None	Treatment with HCARE leads to significantly higher improvement, relative to placebo, in the FSFI Total score (p<0.001), FSFI domain score for "arousal" (p<0.001), "lubrication" (p<0.001), "orgasm" (p = 0.004), and "satisfaction" (p<0.001), and also FSDS score (p<0.001) and the number of successful sexual encounters (p<0.001) at the end of the treatment

Limitations

This review on the potential benefits of these Ayurvedic herbs in reproductive health, while insightful, has several inherent limitations that should be considered. The diversity in study designs, ranging from animal experiments to human trials, introduced challenges in making direct comparisons and drawing universally applicable conclusions. Some studies featured small sample sizes, limiting the generalizability of findings to broader populations. The variation in study durations raised questions about the long-term effects and potential side effects of the interventions explored. The psychological aspect of placebo effects, as observed in some studies, underscored the need for a cautious interpretation of subjective outcomes. Confounding factors, such as co-administration with other substances or interventions, may have complicated the interpretation of observed effects. These factors indicated the need for additional well-designed, long-term future research to enhance the robustness and generalizability of findings in the realm of women’s reproductive health.

## Conclusions

The resilience of Ayurveda lies not only in its historical legacy but also in its adaptability to contemporary needs. This review of available data regarding the usage of various Ayurvedic herbs in female reproductive health underscores the need for a paradigm shift as the global community grapples with the challenges posed by conventional medicine's limitations and side effects. In light of the presented studies, *Asparagus racemosus* shows promise in addressing conditions such as dysmenorrhea, lactation, and infertility, with effects comparable to clomiphene citrate in stimulating follicular growth. Tulsi extract also appears to be comparable to clomiphene citrate in inducing ovulation in PCOS, while its potential for usage as a contraceptive requires further study. Cardamom shows efficacy in treating nausea in mothers undergoing spinal anesthesia and in the first trimester, and in managing PCOS. Among the herbs reviewed, ginger appeared to have the widest range of applications, with the potential for therapeutic management of nausea and vomiting during pregnancy, infertility, PCOS, and dysmenorrhea. Both ginger and ashwagandha also exhibited promising anticarcinogenic properties, particularly in the context of ovarian and endometrial cancers. The diverse applications of these herbs call for the integration of natural, personalized medicine in patient care. Ayurveda is not only an exploration of the past but a quest into a future where comprehensive care stands as the cornerstone of medicine.

## References

[1] Mukherjee PK, Nema NK, Venkatesh P, Debnath PK (2012). Changing scenario for promotion and development of Ayurveda--way forward. J Ethnopharmacol.

[2] Gyawali D, Vohra R, Orme-Johnson D, Ramaratnam S, Schneider RH (2021). A systematic review and meta-analysis of Ayurvedic herbal preparations for hypercholesterolemia. Medicina (Kaunas).

[3] Pandey MM, Rastogi S, Rawat AK (2013). Indian traditional ayurvedic system of medicine and nutritional supplementation. Evid Based Complement Alternat Med.

[4] Patel M, Vishnoi S, Neelima A (2023). An empirical review of fundamental principles of Ayurveda for women’s reproductive health and diseases. J Ayurveda Integr Med Sci.

[5] Palatty PL, Haniadka R, Valder B, Arora R, Baliga MS (2013). Ginger in the prevention of nausea and vomiting: a review. Crit Rev Food Sci Nutr.

[6] Hasan N, Ahmad N, Zohrameena Zohrameena, Khalid M, Akhtar J (2016). Asparagus racemosus: for medicinal uses & pharmacological actions. Int J Adv Res.

[7] Joshi RK (2016). Asparagus racemosus (Shatawari), phytoconstituents and medicinal importance, future source of economy by cultivation in Uttrakhand: a review. Int J Herb Med.

[8] Singh R (2016). Asparagus racemosus: a review on its phytochemical and therapeutic potential. Nat Prod Res.

[9] Kaaria LM, Oduma JA, Kaingu CK, Mutai PC, Wafula DK (2019). Effect of Asparagus racemosus on selected female reproductive parameters using Wistar rat model. J Nat Prod Res Ethnopharmacol.

[10] Kumar S, Mehla RK, Singh M (2014). Effect of Shatavari (Asparagus racemosus) on milk production and immune-modulation in Karan Fries crossbred cows. Indian J Tradit Knowl.

[11] Gupta M, Shaw B (2011). A double-blind randomized clinical trial for evaluation of galactogogue activity of Asparagus racemosus willd. Iran J Pharm Res.

[12] Majeedi S, Shameem I, Roqaiya M (2016). Efficacy of Asparagus recemosus (Satavar) in stimulating follicular growth and ovulation in anovulatory infertility: a randomized controlled trial. Int J Reprod Contracept Obstet Gynecol.

[13] Farzana MUZN, Sultana A (2015). Clinical Study of Glycyrrhiza glabra Linn and Asparagus racemosus Linn on Menopausal Symptoms. https://www.researchgate.net/publication/343136443_Clinical_Study_of_Glycyrrhiza_glabra_Linn_and_Asparagus_racemosus_Linn_on_Menopausal_Symptoms.

[14] Bhandary BSH, Sharmila KP, Kumari NS, Bhat VS, Fernandes R (2017). Acute and subacute toxicity profile of Asparagus racemosus root extract, isoprinosine and Shatvari syrup in Swiss albino mice. J App Pharm Sci.

[15] Sharma N, Kumar V, Langeh U, Singh C, Singh A (2023). A review on the pharmacological potential of Indian spices in polycystic ovarian syndrome. J Reprod Health Med.

[16] Sengupta A, Bhattacharjee S (2009). Cardamom (Elettaria cardamomum) and its active constituent, I,8-cineole. Mol Targets Ther Uses Spices.

[17] Abdullah Abdullah, Ahmad N, Tian W (2022). Recent advances in the extraction, chemical composition, therapeutic potential, and delivery of cardamom phytochemicals. Front Nutr.

[18] Sari AJP, Damanik RA, Gultom YT (2023). The effect of giving Kajah pudding (cardamom ginger) on reducing the frequency of nausea and vomiting in first trimester pregnant women in the Medan Tuntungan Health Center working area in 2023. J Edu Health.

[19] Lete I, Allué J (2016). The effectiveness of ginger in the prevention of nausea and vomiting during pregnancy and chemotherapy. Integr Med Insights.

[20] Khatiban M, Mirzaie M, Fazeli A, Tapak L, Khalili Z (2022). Effect of cardamom inhalation therapy on intra-and postoperative nausea and vomiting of mothers undergoing spinal anesthesia for elective cesarean section. J Perianesth Nurs.

[21] Cheshmeh S, Ghayyem M, Khamooshi F (2022). Green cardamom plus low-calorie diet can decrease the expression of inflammatory genes among obese women with polycystic ovary syndrome: a double-blind randomized clinical trial. Eat Weight Disord.

[22] Cheshmeh S, Elahi N, Ghayyem M, Mosaieby E, Moradi S, Pasdar Y, Tahmasebi S (2021). Effects of green cardamom supplementation on obesity and diabetes gene expression among obese women with polycystic ovary syndrome; a double blind randomized controlled trial [PREPRINT]. ResearchSquare.

[23] Masoumi-Ardakani Y, Mandegary A, Esmaeilpour K, Najafipour H, Sharififar F, Pakravanan M, Ghazvini H (2016). Chemical composition, anticonvulsant activity, and toxicity of essential oil and methanolic extract of Elettaria cardamomum. Planta Med.

[24] Sirotkin AV (2022). The influence of turmeric and curcumin on female reproductive processes. Planta Med.

[25] Kamal DA, Salamt N, Yusuf AN, Kashim MI, Mokhtar MH (2021). Potential health benefits of curcumin on female reproductive disorders: a review. Nutrients.

[26] Cao H, Wei YX, Zhou Q, Zhang Y, Guo XP, Zhang J (2017). Inhibitory effect of curcumin in human endometriosis endometrial cells via downregulation of vascular endothelial growth factor. Mol Med Rep.

[27] Swarnakar S, Paul S (2009). Curcumin arrests endometriosis by downregulation of matrix metalloproteinase-9 activity. Indian J Biochem Biophys.

[28] Gudarzi R, Shabani F, Mohammad-Alizadeh-Charandabi S, Naghshineh E, Shaseb E, Mirghafourvand M (2024). Effect of curcumin on painful symptoms of endometriosis: a triple-blind randomized controlled trial. Phytother Res.

[29] Shah MZ, Shrivastava VK (2022). Turmeric extract alleviates endocrine-metabolic disturbances in letrozole-induced PCOS by increasing adiponectin circulation: a comparison with metformin. Metabol Open.

[30] Sohrevardi SM, Heydari B, Azarpazhooh MR (2021). Therapeutic effect of curcumin in women with polycystic ovary syndrome receiving metformin: a randomized controlled trial. Adv Exp Med Biol.

[31] Asan S, Baş M, Eren B, Karaca E (2020). The effects of curcumin supplementation added to diet on anthropometric and biochemical status in women with polycystic ovary syndrome: a randomized, placebo-controlled trial. Prog Nutr.

[32] Dyawapur A, Patil NG, Metri L (2018). Effectiveness of cinnamon tea and turmeric water for reducing dysmenorrhoea among degree girls. Int J Sci Healthc Res.

[33] Okuyan E, Gunakan E, Atac H, Cakmak Y (2021). The effect of turmeric on primary dysmenorrhea: prospective case-control study. J Surg Med.

[34] Bahrami A, Zarban A, Rezapour H, Agha Amini Fashami A, Ferns GA (2021). Effects of curcumin on menstrual pattern, premenstrual syndrome, and dysmenorrhea: a triple-blind, placebo-controlled clinical trial. Phytother Res.

[35] Soleimani V, Sahebkar A, Hosseinzadeh H (2018). Turmeric (Curcuma longa) and its major constituent (curcumin) as nontoxic and safe substances: Review. Phytother Res.

[36] Tossetta G, Fantone S, Giannubilo SR, Marzioni D (2021). The multifaced actions of curcumin in pregnancy outcome. Antioxidants (Basel).

[37] Sethi L, Bhadra P (2020). A review paper on Tulsi plant (Ocimum sanctum L.). Indian J Nat Sci.

[38] Sahoo DD, Tabassum Y, Sharma D (2022). Multiple health benefits of Tulsi plants. J Med Plants Stud.

[39] Poli V, Challa C (2019). A comparative study of eugenol and Ocimum sanctum Linn. leaf extract on the antifertility effect in female albino rats. J Chin Med Assoc.

[40] Poli V, Reddy MS (2020). The potential role of eugenol and Ocimum sanctum extract on female rats: a focus on infertility efficacy. Iraq Med J.

[41] Farhana A, Reddy TA, Bhavana K, Mutha S, Bakshi V (2018). Assessment of Ocimum sanctum to normalize the estrous cycle in letrozole induced polycystic ovary syndrome in female Wistar rats. World J Pharm Res.

[42] Raina P, Chandrasekaran CV, Deepak M, Agarwal A, Ruchika KG (2015). Evaluation of subacute toxicity of methanolic/aqueous preparation of aerial parts of O. sanctum in Wistar rats: clinical, haematological, biochemical and histopathological studies. J Ethnopharmacol.

[43] Akimoto M, Iizuka M, Kanematsu R, Yoshida M, Takenaga K (2015). Anticancer effect of ginger extract against pancreatic cancer cells mainly through reactive oxygen species-mediated autotic cell death. PLoS One.

[44] Li N, Xing Y, Sultan AH, Raeeszadeh M, Akbari A, Liu H (2021). Ginger (Zingiber officinale roscoe) improves ethanol-induced reproductive dysfunction by enhancing steroidogenesis and inhibiting oxidative stress and inflammation. Braz Arch Biol Technol.

[45] Choi SH, Shapiro H, Robinson GE (2005). Psychological side-effects of clomiphene citrate and human menopausal gonadotrophin. J Psychosom Obstet Gynaecol.

[46] Yılmaz N, Seven B, Timur H (2018). Ginger (zingiber officinale) might improve female fertility: a rat model. J Chin Med Assoc.

[47] Usman AN, Raya I, Yasmin R (2021). Ginger honey affects cortisol, estrogen and glutathione levels; preliminary study to target preconceptional women. Gac Sanit.

[48] Atashpour S, Kargar Jahromi H, Kargar Jahromi Z, Maleknasab M (2017). Comparison of the effects of ginger extract with clomiphene citrate on sex hormones in rats with polycystic ovarian syndrome. Int J Reprod Biomed.

[49] Bonab SB (2020). The effect of 12-week pilates training and ginger supplementation on polycystic ovary syndrome in women. Studies Med Sci.

[50] Rhode J, Fogoros S, Zick S, Wahl H, Griffith KA, Huang J, Liu JR (2007). Ginger inhibits cell growth and modulates angiogenic factors in ovarian cancer cells. BMC Complement Altern Med.

[51] Naveed M, Imran M, Khalid S, Qureshi I, Ahmad I, Inayat S, Imtiaz F (2022). Comparative effect of ginger and vitamin E supplements on pain and quality of life among females with dysmenorrhea: a randomized controlled trial. Pak Biomed J.

[52] Harjai K, Chand R (2018). A study to assess the effectiveness of ginger for reducing pain in primary dysmenorrhoea among adolescent girls in selected college of nursing at Dehradun. Int J Res Cult Soc.

[53] Kim SD, Kwag EB, Yang MX, Yoo HS (2022). Efficacy and safety of ginger on the side effects of chemotherapy in breast cancer patients: systematic review and meta-analysis. Int J Mol Sci.

[54] Viljoen E, Visser J, Koen N, Musekiwa A (2014). A systematic review and meta-analysis of the effect and safety of ginger in the treatment of pregnancy-associated nausea and vomiting. Nutr J.

[55] Thomson M, Corbin R, Leung L (2014). Effects of ginger for nausea and vomiting in early pregnancy: a meta-analysis. J Am Board Fam Med.

[56] Lopresti AL, Smith SJ, Malvi H, Kodgule R (2019). An investigation into the stress-relieving and pharmacological actions of an ashwagandha (Withania somnifera) extract: a randomized, double-blind, placebo-controlled study. Medicine (Baltimore).

[57] Kulkarni SK, Dhir A (2008). Withania somnifera: an Indian ginseng. Prog Neuropsychopharmacol Biol Psychiatry.

[58] Mirjalili MH, Moyano E, Bonfill M, Cusido RM, Palazón J (2009). Steroidal lactones from Withania somnifera, an ancient plant for novel medicine. Molecules.

[59] Rahi S, Gupta HP, Prasad S, Baithalu RK (2013). Phytotherapy for endometritis and subsequent conception rate in repeat breeding crossbred cows. Indian J Anim Reprod.

[60] Gopal S, Ajgaonkar A, Kanchi P, Kaundinya A, Thakare V, Chauhan S, Langade D (2021). Effect of an ashwagandha (Withania Somnifera) root extract on climacteric symptoms in women during perimenopause: a randomized, double-blind, placebo-controlled study. J Obstet Gynaecol Res.

[61] Xu K, Shi H, Du Y, Ou J (2021). Withaferin A inhibits proliferation of human endometrial cancer cells via transforming growth factor-β (TGF-β) signalling. 3 Biotech.

[62] Dongre S, Langade D, Bhattacharyya S (2015). Efficacy and safety of Ashwagandha (Withania somnifera) root extract in improving sexual function in women: a pilot study. Biomed Res Int.

[63] Verma N, Gupta SK, Tiwari S, Mishra AK (2021). Safety of Ashwagandha root extract: A randomized, placebo-controlled, study in healthy volunteers. Complement Ther Med.

